# Mechanical ventilation core outcome set uptake in Cochrane systematic reviews: A cross‐sectional study

**DOI:** 10.1002/cesm.12038

**Published:** 2024-01-10

**Authors:** Luis Garegnani, Diego Ivaldi, Mariana Burgos, Gisela Oltra, Camila M. Escobar Liquitay

**Affiliations:** ^1^ Research Department Instituto Universitario Hospital Italiano de Buenos Aires Buenos Aires Argentina

**Keywords:** core outcome set, mechanical ventilation, patients' values and preferences, systematic reviews

## Abstract

**Introduction:**

The Cochrane Handbook acknowledges core outcome sets' (COS) relevance in defining review questions and planning systematic reviews. We aimed to assess the uptake of the mechanical ventilation (MV) COS in Cochrane systematic reviews of interventions.

**Methods:**

A cross‐sectional study. We included Cochrane systematic reviews and protocols of Cochrane systematic reviews of any intervention related to mechanically ventilated patients through a search in the Cochrane database of systematic reviews. We did not apply restrictions based on age or setting. One reviewer assessed the studies for eligibility and extracted data. Both processes were validated by a second author.

**Results:**

We identified 233 reviews and protocols through our search strategy. We finally included 36 records. Thirty‐four (94.44%) were Cochrane reviews and two (5.56%) were protocols. The included Cochrane reviews and protocols assessed a median of 13 (interquartile range [IQR]: 9–17) outcomes, with 35 (97.22%) reviews reporting at least one outcome from the MV COS. The median number of outcomes from the MV COS reported in the Cochrane reviews and protocols was 2.5 (IQR: 2–3). Only one (2.78%) study reported all the outcomes from the MV COS. None of the included Cochrane reviews and protocols cited the MV COS publication.

**Conclusion:**

Completed Cochrane systematic reviews and protocols of Cochrane systematic reviews of interventions related to mechanically ventilated patients have an overall limited uptake of the MV COS so far. Mortality and duration of stay were the most reported outcomes, while extubation and reintubation were the least informed. These findings may serve as a starting point for the Cochrane Critical Care community to develop dissemination strategies for improving the MV COS uptake.

## 1 INTRODUCTION

Core outcome sets (COS) is “an agreed standardized set of outcomes that should be measured and reported, as a minimum, in all clinical trials in specific areas of health or health care” by the Core Outcome Measures for Effectiveness Trials Initiative [1, 2]. They help reduce research waste by improving the consistency of outcomes measured in trials and other research of the same health condition, involving critical stakeholders and consensus methods, ensuring that all essential outcomes are measured, and reducing outcome reporting bias [3]. COS usually consists of a combination of “domains,” a specific “area” which should be measured (e.g., quality of life), and “instruments,” which are the outcome measure for that particular domain (e.g., questionnaires to assess the quality of life) [4].

Systematic reviews of interventions summarize all the available evidence on a given topic and are used to inform healthcare decision‐making [5]. Clinical practice guideline (CPG) developing panels should also use systematic reviews to produce reliable CPG [6]. Cochrane reviews support almost 70% of recommendations in Latin American CPGs and 28% of Danish CPGs' recommendations [7, 8]. Frequently, the choice of outcomes for a systematic review raises concerns because clinical trialists and systematic reviewers are interested in different outcomes, precluding studies from being included in the systematic review analysis [5]. Cochrane reviews are well known for their rigorous methods and high‐quality standards, with the relevance of COS in defining review questions and planning the review, acknowledged in the *Cochrane Handbook for Systematic Reviews of Interventions* [9]. Furthermore, the structure of Cochrane reviews was updated in 2023 to ensure that Cochrane authors include the COS outcomes in their review [9], encouraging to search and incorporate COS and changing the outcome classification from “primary” and “secondary” outcomes to “critical” and “important” outcomes, among other new features [9].

Several Cochrane Reviews evaluating mechanical ventilation (MV) strategies and weaning protocols spanning 2 decades highlighted substantial variation in outcome selection [10, 11], leading to the development of a COS for trials testing any intervention intended to modify MV duration in critical care [12]. This particular COS also aimed to engage relevant participants in identifying important core outcomes for these trials and to obtain agreement on how they should be defined, measured and reported [12].

Although this particular COS has been available for a short period of time since its final publication in 2019, which may limit the implementation and application in primary and secondary research, its uptake in systematic reviews still needs to be assessed as a starting point for monitoring and evaluation.

## 2 OBJECTIVE

To assess the uptake of the MV COS in Cochrane systematic reviews of interventions. We hypothesize that MV COS uptake in Cochrane systematic reviews of interventions is low.

## 3 METHODS

A cross‐sectional study. The study methods were compliant with the STrengthening the Reporting of OBservational studies in Epidemiology checklist [13] and the guidelines for reporting meta‐epidemiological methodology research [14].

We included completed Cochrane systematic reviews of interventions or protocols of Cochrane systematic reviews of intervention published since January 1, 2020, based on the publication date of the MV COS (October 2019). We included reviews or protocols assessing the effect of pharmacological interventions (like corticosteroids, blood transfusions, of antimicrobials therapies), nonpharmacological interventions (like mobilization or exercise therapy, MV setting strategies) or more complex intervention like the combination of pharmacological and nonpharmacological interventions. We included reviews or protocols in which only a subset of participants requiered MV, independently if data was available separately for this subset. Although the MV COS was developed for adults in critical care, we did not apply restrictions based on age or setting, trying to capture the broader scope possible. Furthermore, there is no consensus on the definition of “critical care,” as some definitions include identification, monitoring, and treatment of patients with critical illness through the initial and sustained support of vital organ functions. Critical care includes both high‐resource care in intensive care units and lower resource care in other settings, as it can be provided in general wards, in small health facilities, in the community or in ambulances [15].

We searched the Cochrane database of systematic reviews, with the last date of search in February 2023. For the detailed search strategy, see File S1: Appendix.

One reviewer (out of D. I., M. B., G. O., and C. M. E. L.) assessed the retrieved studies for eligibility, validated by the lead author (L. G.). For reviews fulfilling our inclusion criteria, we extracted key information on participants, interventions, comparators, and reported outcomes from the MV COS using a specific spreadsheet developed for this research. We also extracted data related to outcomes not included in the MV COS. One reviewer (out of D. I., M. B., G. O., and C. M. E. L.) extracted data, and the lead author (L. G.) validated it. We solved disagreements in data extraction through discussion.

We assessed the frequency of Cochrane systematic reviews and protocols reporting each component of the COS. These were extubation, reintubation, duration of MV, duration of stay (both Intensive care unit and hospital stay), and quality of life, defined according to Blackwood et al. We also analyzed the MV COS citation by inspecting the reference list.

We reported continuous variables as means and standard deviations or medians and interquartile ranges according to the distribution, analyzed by visual inspection of histograms, standardized normal probability plots, and the Shapiro–Wilk test. We reported categorical variables as absolute numbers and proportions. We used STATA 16.0 software for the statistical analysis (StataCorp LLC) [16].

## 4 RESULTS

**Figure 1:**
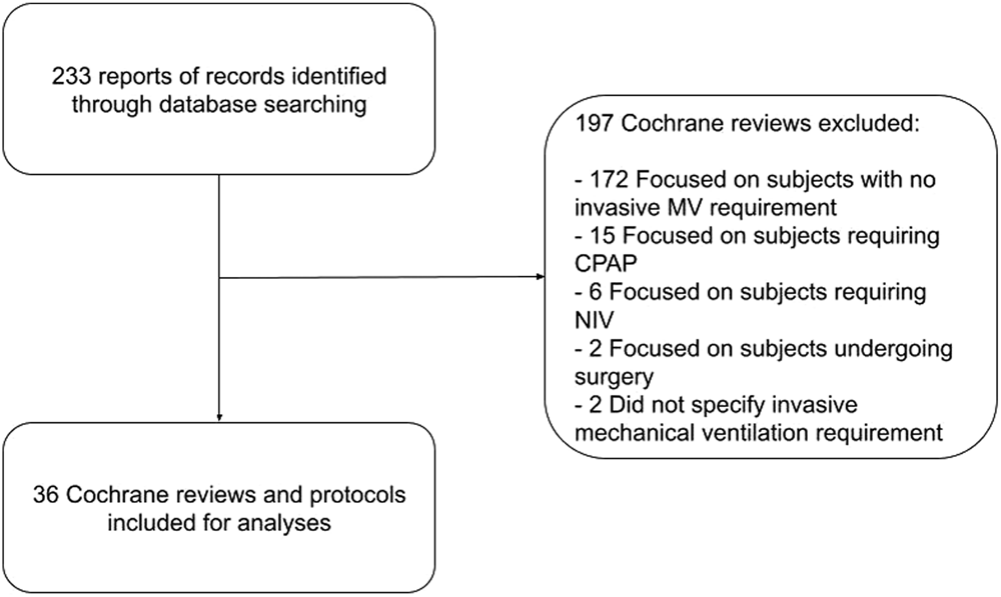
Study flow diagram. CPAP, continuous positive airway pressure; MV, mechanical ventilation; NIV, noninvasive ventilation.

We identified 233 reviews and protocols. We excluded 197 records because they did not focus on mechanically ventilated patients and finally included 36 records [17, 18, 19, 20, 21, 22, 23, 24, 25, 26, 27, 28, 29, 30, 31, 32, 33, 34, 35, 36, 37, 38, 39, 40, 41, 42, 43, 44, 45, 46, 47, 48, 49, 50, 51]. See the flow diagram in Figure 1. See the list and characteristics of excluded studies in File S1: Appendix.

Of the 36 included records, 34 (94.44%) were completed Cochrane reviews and 2 (5.56%) were protocols. A full description of the included Cochrane reviews and protocols is available in Table 1.

**Table 1:** Characteristics of included studies.

Variable	Descriptive statistics
Year of publication	
2020	10/36 (27.78%)
2021	15/36 (41.67%)
2022	8/36 (22.22%)
2023	3/36 (8.33%)
Population age	
Adults	14/36 (38.89%)
Children	16/36 (44.44%)
No age restriction	6/36 (16.67%)
Specific population	
MV participants only	15/36 (41.67%)
Mixed population	21/36 (58.33%)
Health condition	
ARDS	7/36 (19.44%)
Airway disease	2/36 (5.56%)
COVID‐19	8/36 (22.22%)
Critical illness, in general	7/36 (19.44%)
Guillain–Barré syndrome	1/36 (2.78%)
Heart failure	1/36 (2.78%)
Gastrointestinal disease	3/36 (8.33%)
Preterm infants	4/36 (1.11%)
Other pulmonary diseases	2/36 (5.56%)
Surgery	1/36 (2.78%)
Cochrane Review Group	
Cochrane Acute Respiratory Infections Group	3/36 (8.33%)
Cochrane Consumers and Communication Group	1/36 (2.78%)
Cochrane Emergency and Critical Care Group	4/36 (11.11%)
Cochrane Gut Group	2/36 (5.56%)
Cochrane Haematology Group	7/36 (19.44%)
Cochrane Heart Group	1/36 (2.78%)
Cochrane Kidney and Transplant Group	1/36 (2.78%)
Cochrane Neonatal Group	15/36 (41.67%)
Cochrane Neuromuscular Group	1/36 (2.78%)
Cochrane Oral Health Group	1/36 (2.78%)

Abbreviations: ARDS, acute respiratory distress syndrome; COVID‐19, coronavirus disease of 2019; MV, mechanical ventilation.

Cochrane reviews including mixed populations did not report the proportion of MV participants in the analyses of the included studies. Interventions considered in the reviews were highly variable, with 20 (55.56%) reviews assessing pharmacological interventions, 15 (41.67%) reviews evaluating nonpharmacological interventions, and 1 (2.78%) review assessing both pharmacological and nonpharmacological interventions. See additional information about assessed interventions in Table 2.

**Table 2:** Examples of interventions considered in Cochrane reviews and protocols by CRG.

CRG	Interventions
Cochrane Acute Respiratory Infections Group	Body positioning, topical antibiotic prophylaxis, chest physiotherapy
Cochrane Consumers and Communication Group	Communication aids
Cochrane Emergency and Critical Care Group	High PEEP, early spontaneous breathing, IL‐6 blocking agents, neuromuscular blocking agents
Cochrane Gut Group	Monitoring gastric residual volume, electromagnetic‐guided postpyloric placement of nasoenteral feeding tubes
Cochrane Haematology Group	Convalescent plasma, systemic corticosteroids, Janus kinase inhibitor, remdesivir, colchicine, hyperimmune animal sera, any antibiotic
Cochrane Heart Group	Prophylactic corticosteroids
Cochrane Kidney and Transplant Group	Drugs for heart failure
Cochrane Neonatal Group	Any PEEP level, CPAP, lung recruitment maneuvers, sustained (>1 s) inflation, opioids, flexible thin catheter for surfactant therapy, systemic corticosteroids, inhaled corticosteroids, surfactant, sound reduction, cuffed endotracheal tubes, push gavage feeding, early parenteral nutrition, corticosteroids‐surfactant mixture
Cochrane Neuromuscular Group	Pharmacological treatments other than corticosteroids
Cochrane Oral Health Group	Oral hygiene care procedures with pharmacological agents and different types of tooth‐brushing

Abbreviations: CPAP, continuous positive airway pressure; CRG, Cochrane Review Group; IL, interleukin; PEEP, positive end‐expiratory pressure.

The included Cochrane reviews and protocols assessed a median of 13 (interquartile range [IQR]: 9–17) outcomes, with 35 (97.22%) reviews reporting at least one outcome from the MV COS. The median number of outcomes from the MV COS reported in the Cochrane reviews and protocols was 2.5 (IQR: 2–3). Only one (2.78%) review reported all the outcomes from the MV COS. None of the included Cochrane reviews and protocols cited the publication of the MV COS by Blackwood et al. [12]. See Table 3 for detailed information about MV COS uptake in the included studies.

**Table 3:** Uptake of the MV COS set in cochrane systematic reviews and protocols.

MV COS	All Cochrane reviews, *n* (%)	MV participants only, *n* (%)	Mixed population, *n* (%)
Extubation	3/36 (8.33%)	2/15 (13.33%)	1/21 (3.33%)
Reintubation	3/36 (8.33%)	2/15 (13.33%)	1/21 (3.33%)
Duration of MV	15/36 (41.67%)	8/15 (53.33%)	7/21 (23.33%)
Duration of stay	24/36 (66.67%)	12/15 (80.00%)	12/21 (40.00%)
Mortality	33/36 (91.67%)	15/15 (100%)	18/21 (60.00%)
Quality of life	10/36 (27.78%)	2/15 (13.33%)	8/21 (26.67%)

Abbreviations: COS, core outcome set; MV, mechanical ventilation.

In an exploratory analysis, studies focusing exclusively on adults were more likely to report quality of life (odds ratio [OR]: 13.22, 95% confidence interval [CI]: 1.79–148.12, *p* = 0.0017). We did not find a similar trend with other outcomes from the MV COS. We also found that reviews focusing exclusively on mechanically ventilated patients were not associated with reporting any individual outcome from the COS. Additionally, we found that reviews focusing on nonpharmacological interventions were more likely to report duration of stay (OR: 7, 95% CI: 1.07–75.22, *p* = 0.0177), but did not find a similar trend with other MV COS outcomes.

The included Cochrane reviews assessed a median of 10 (IQR: 6–14.25) outcomes not included in the MV COS. These outcomes were highly variable, ranging from oxygen saturation, blood gases, lung function, and respiratory mechanics to adverse events‐related outcomes, clinical deterioration, or hospital‐acquired infections.

## 5 DISCUSSION

Our findings show that Cochrane systematic reviews and protocols of any intervention related to mechanically ventilated patients have an overall limited uptake of the MV COS developed by Blackwood et al. [12]. This is not surprising, as the development of this COS was the first step toward an agreed set of outcomes by the critical care community. Other healthcare fields, like rheumatology, have a longer tradition in developing, disseminating, implementing and assessing the uptake of several COS [52, 53, 54]. Although none of the included reviews cited the MV COS paper, almost all studies incorporated at least one outcome from the MV COS. Mortality and duration of stay were the most reported outcomes, while extubation and reintubation were the least informed. Only one review reported all the outcomes from the MV COS.

A recent study assessing the use of existing COS to inform the choice of outcomes in Cochrane systematic reviews [5] found that less than 10% of the reviews referenced a COS about the choice of outcomes. Although it had a broader approach, not restricted to reviews focusing on MV participants, their results align with the low COS citation found in our study.

Several studies assessed COS uptakes [55, 56, 57, 58, 59] in clinical trials and systematic reviews across different health areas and found wide variation in COS uptake. Overall, the studies found low COS uptake levels, ranging from 20% to 45%. Implemented methods to evaluate COS uptake in these studies were also highly variable, using several approaches like citation analysis or outcomes extraction from registers or protocols, as conducted in our research [1, 60].

Studies focusing on other health conditions, like rheumatoid arthritis [61], showed an exceptionally high rate of COS uptake, suggesting that COS endorsement by scientific societies or regulatory agencies might impact COS uptake. However, this issue still needs to be addressed and may require additional research.

Our study is not free from limitations. First, we followed a broad approach to assessing the outcomes reported in Cochrane reviews and protocols to maximize the use of available information and show the situation diagnosis in outcome selection for people requiring MV, imposing no age limits for study eligibility. Although the MV COS is valid for randomized trials of interventions for invasively mechanically ventilated adults in critical care, it considered adults and children at the protocol stage [62]. Yet, no results are available for children. Second, we did not apply any specific criteria for intervention eligibility. This may have led us to include reviews assessing interventions with limited effects on MV patients and outcomes included in the COS. Third, noticing that Cochrane reviews or protocols take almost 2 years for publication [63], we considered a short time frame for assessing the MV COS uptake only 3–4 years after its publication. Nevertheless, our findings may serve as a starting point for the Cochrane Critical Care community to develop dissemination strategies for improving the MV COS uptake among authors, editors and the Cochrane community members in general, with a special focus on groups developing reviews that did not follow the MV COS.

Further studies focusing on the uptake of the MV COS in primary studies are needed. Both trialists and systematic reviewers need to consider established COS when available for specific health conditions when selecting outcomes for their research.

## 6 CONCLUSION

Cochrane systematic reviews and protocols of Cochrane systematic reviews of interventions related to mechanically ventilated patients have an overall limited uptake of the MV COS so far. Mortality and duration of stay were the most reported outcomes, while extubation and reintubation were the least informed. These findings may serve as a starting point for the Cochrane Critical Care community to develop dissemination strategies for improving the MV COS uptake.

## AUTHOR CONTRIBUTIONS

**Luis Garegnani**: Conceptualization; methodology; validation; formal analysis; investigation; writing; visualization; supervision; project administration. **Diego Ivaldi**: Conceptualization; formal analysis. **Mariana Burgos**: Data curation; formal analysis; investigation; writing—original draft. **Gisela Oltra**: Data curation; investigation; methodology. **Camila M. Escobar Liquitay**: Data curation; formal analysis; investigation; methodology; project administration.

## CONFLICT OF INTEREST STATEMENT

The authors declare no conflict of interest.

## PEER REVIEW

The peer review history for this article is available at https://www.webofscience.com/api/gateway/wos/peer-review/10.1002/cesm.12038.

## Supporting information

Supporting information.

## Data Availability

The data that support the findings of this study are available on request from the corresponding author.
