# Supplementary material for: Mechanical ventilation core outcome set uptake in Cochrane systematic reviews: A cross‐sectional study
Source: Cochrane Evid Synth Methods. 2024 Jan 10;2(1):e12038. doi: 10.1002/cesm.12038 (PMC11795963; doi:10.1002/cesm.12038)
Supplement: Supplementary file 1 — Supporting information. [file CESM-2-e12038-s001.docx]

**Mechanical ventilation core outcome set uptake in Cochrane systematic reviews. A cross-sectional study**

**Appendix**

**Search strategy**

#1 MeSH descriptor: [Respiration, Artificial] explode all trees

#2 (Artificial Respiration*):ti,ab,kw

#3 (Mechanical Ventilation*):ti,ab,kw

#4 (Positive Pressure Respiration*):ti,ab,kw

#5 (ventilation*):ti,ab,kw

#6 (respirator*):ti,ab,kw

#7 #1 OR #2 OR #3 OR #4 OR #5 OR #6

Date of publication: 01/01/2020 to 22/02/2023

Intervention

**Characteristics of excluded studies**

| **Title** | **DOI** | **Full review/Protocol** | **Detailed Reason for exclusion** |
| --- | --- | --- | --- |
| Non‐invasive respiratory support for the management of transient tachypnea of the newborn | 10.1002/14651858.CD013231.pub2 | Full review | Focused on subjects requiering NIV |
| Postnatal corticosteroids for transient tachypnoea of the newborn | 10.1002/14651858.CD013222.pub2 | Full review | Focused on subjects with no invasive mechanical ventilation requierement |
| Diaphragm‐triggered non‐invasive respiratory support in preterm infants | 10.1002/14651858.CD012935.pub2 | Full review | Focused on subjects with no invasive mechanical ventilation requierement |
| Myofunctional therapy (oropharyngeal exercises) for obstructive sleep apnoea | 10.1002/14651858.CD013449.pub2 | Full review | Focused on subjects with no invasive mechanical ventilation requierement |
| Educational, supportive and behavioural interventions to improve usage of continuous positive airway pressure machines in adults with obstructive sleep apnoea | 10.1002/14651858.CD007736.pub3 | Full review | Focused on subjects with no invasive mechanical ventilation requierement |
| Oscillating devices for airway clearance in people with cystic fibrosis | 10.1002/14651858.CD006842.pub5 | Full review | Focused on subjects with no invasive mechanical ventilation requierement |
| Magnesium sulphate for treating acute bronchiolitis in children up to two years of age | 10.1002/14651858.CD012965.pub2 | Full review | Focused on subjects with no invasive mechanical ventilation requierement |
| Oxygen therapy in the pre‐hospital setting for acute exacerbations of chronic obstructive pulmonary disease | 10.1002/14651858.CD005534.pub3 | Full review | Focused on subjects with no invasive mechanical ventilation requierement |
| Early versus delayed continuous positive airway pressure (CPAP) for respiratory distress in preterm infants | 10.1002/14651858.CD002975.pub2 | Full review | Focused on subjects requiering CPAP |
| Interventions for promoting physical activity in people with chronic obstructive pulmonary disease (COPD) | 10.1002/14651858.CD012626.pub2 | Full review | Focused on subjects with no invasive mechanical ventilation requierement |
| Interventions to reduce contaminated aerosols produced during dental procedures for preventing infectious diseases | 10.1002/14651858.CD013686.pub2 | Full review | Focused on subjects with no invasive mechanical ventilation requierement |
| Antimicrobial mouthwashes (gargling) and nasal sprays administered to patients with suspected or confirmed COVID‐19 infection to improve patient outcomes and to protect healthcare workers treating them | 10.1002/14651858.CD013627.pub2 | Full review | Focused on subjects with no invasive mechanical ventilation requierement |
| Antimicrobial mouthwashes (gargling) and nasal sprays to protect healthcare workers when undertaking aerosol‐generating procedures (AGPs) on patients without suspected or confirmed COVID‐19 infection | 10.1002/14651858.CD013628.pub2 | Full review | Focused on subjects with no invasive mechanical ventilation requierement |
| Drug treatments for managing cystic fibrosis‐related diabetes | 10.1002/14651858.CD004730.pub5 | Full review | Focused on subjects with no invasive mechanical ventilation requierement |
| Clonidine for pain in non‐ventilated infants | 10.1002/14651858.CD013104.pub2 | Full review | Focused on subjects with no invasive mechanical ventilation requierement |
| Enteral lactoferrin supplementation for prevention of sepsis and necrotizing enterocolitis in preterm infants | 10.1002/14651858.CD007137.pub6 | Full review | Focused on subjects with no invasive mechanical ventilation requierement |
| Setting and techniques for monitoring blood pressure during pregnancy | 10.1002/14651858.CD012739.pub2 | Full review | Focused on subjects with no invasive mechanical ventilation requierement |
| Personal protective equipment for preventing highly infectious diseases due to exposure to contaminated body fluids in healthcare staff | 10.1002/14651858.CD011621.pub5 | Full review | Focused on subjects with no invasive mechanical ventilation requierement |
| Combination antimicrobial susceptibility testing for acute exacerbations in chronic infection of Pseudomonas aeruginosa in cystic fibrosis | 10.1002/14651858.CD006961.pub5 | Full review | Focused on subjects with no invasive mechanical ventilation requierement |
| Mobility management to prevent, reduce, or delay driving a car in teenagers | 10.1002/14651858.CD009438.pub2 | Full review | Focused on subjects with no invasive mechanical ventilation requierement |
| Adalimumab for maintenance of remission in Crohn's disease | 10.1002/14651858.CD012877.pub2 | Full review | Focused on subjects with no invasive mechanical ventilation requierement |
| Strategies for optimising antenatal corticosteroid administration for women with anticipated preterm birth | 10.1002/14651858.CD013633 | Full review | Focused on subjects with no invasive mechanical ventilation requierement |
| Standard versus biofilm antimicrobial susceptibility testing to guide antibiotic therapy in cystic fibrosis | 10.1002/14651858.CD009528.pub5 | Full review | Focused on subjects with no invasive mechanical ventilation requierement |
| Antibiotic treatment for nontuberculous mycobacteria lung infection in people with cystic fibrosis | 10.1002/14651858.CD010004.pub5 | Full review | Focused on subjects with no invasive mechanical ventilation requierement |
| Home fortification of foods with multiple micronutrient powders for health and nutrition in children under two years of age | 10.1002/14651858.CD008959.pub3 | Full review | Focused on subjects with no invasive mechanical ventilation requierement |
| Timing of hypertonic saline inhalation for cystic fibrosis | 10.1002/14651858.CD008816.pub4 | Full review | Focused on subjects with no invasive mechanical ventilation requierement |
| Probiotics for induction of remission in ulcerative colitis | 10.1002/14651858.CD005573.pub3 | Full review | Focused on subjects with no invasive mechanical ventilation requierement |
| Palliative drug treatments for breathlessness in cystic fibrosis | 10.1002/14651858.CD011855.pub3 | Full review | Focused on subjects with no invasive mechanical ventilation requierement |
| Tonsillectomy versus tonsillotomy for obstructive sleep‐disordered breathing in children | 10.1002/14651858.CD011365.pub2 | Full review | Focused on subjects with no invasive mechanical ventilation requierement |
| Corticosteroids as standalone or add‐on treatment for sore throat | 10.1002/14651858.CD008268.pub3 | Full review | Focused on subjects with no invasive mechanical ventilation requierement |
| Phosphodiesterase‐4 inhibitors for chronic obstructive pulmonary disease | 10.1002/14651858.CD002309.pub6 | Full review | Focused on subjects with no invasive mechanical ventilation requierement |
| Inhaled mannitol for cystic fibrosis | 10.1002/14651858.CD008649.pub4 | Full review | Focused on subjects with no invasive mechanical ventilation requierement |
| Zinc supplementation for the promotion of growth and prevention of infections in infants less than six months of age | 10.1002/14651858.CD010205.pub2 | Full review | Focused on subjects with no invasive mechanical ventilation requierement |
| Rinse‐free hand wash for reducing absenteeism among preschool and school children | 10.1002/14651858.CD012566.pub2 | Full review | Focused on subjects with no invasive mechanical ventilation requierement |
| Omega‐3 fatty acid supplementation for cystic fibrosis | 10.1002/14651858.CD002201.pub6 | Full review | Focused on subjects with no invasive mechanical ventilation requierement |
| Antibiotic treatment for Stenotrophomonas maltophilia in people with cystic fibrosis | 10.1002/14651858.CD009249.pub5 | Full review | Focused on subjects with no invasive mechanical ventilation requierement |
| Physical fitness training for stroke patients | 10.1002/14651858.CD003316.pub7 | Full review | Focused on subjects with no invasive mechanical ventilation requierement |
| Breathing exercises for adults with asthma | 10.1002/14651858.CD001277.pub4 | Full review | Focused on subjects with no invasive mechanical ventilation requierement |
| Probiotics for people with cystic fibrosis | 10.1002/14651858.CD012949.pub2 | Full review | Focused on subjects with no invasive mechanical ventilation requierement |
| Interventions for chronic pruritus of unknown origin | 10.1002/14651858.CD013128.pub2 | Full review | Focused on subjects with no invasive mechanical ventilation requierement |
| Probiotic treatment for women with gestational diabetes to improve maternal and infant health and well‐being | 10.1002/14651858.CD012970.pub2 | Full review | Focused on subjects with no invasive mechanical ventilation requierement |
| Statins for asthma | 10.1002/14651858.CD013268.pub2 | Full review | Focused on subjects with no invasive mechanical ventilation requierement |
| Antibiotic adjuvant therapy for pulmonary infection in cystic fibrosis | 10.1002/14651858.CD008037.pub4 | Full review | Focused on subjects with no invasive mechanical ventilation requierement |
| Anti‐inflammatory medications for obstructive sleep apnoea in children | 10.1002/14651858.CD007074.pub3 | Full review | Focused on subjects with no invasive mechanical ventilation requierement |
| Interventions for preventing and managing advanced liver disease in cystic fibrosis | 10.1002/14651858.CD012056.pub3 | Full review | Focused on subjects with no invasive mechanical ventilation requierement |
| Antibiotic treatment for Burkholderia cepacia complex in people with cystic fibrosis experiencing a pulmonary exacerbation | 10.1002/14651858.CD009529.pub4 | Full review | Focused on subjects with no invasive mechanical ventilation requierement |
| Treatments for the prevention of Sudden Unexpected Death in Epilepsy (SUDEP) | 10.1002/14651858.CD011792.pub3 | Full review | Focused on subjects with no invasive mechanical ventilation requierement |
| Exercise versus no exercise for the occurrence, severity, and duration of acute respiratory infections | 10.1002/14651858.CD010596.pub3 | Full review | Focused on subjects with no invasive mechanical ventilation requierement |
| Vitamin D supplementation for sickle cell disease | 10.1002/14651858.CD010858.pub3 | Full review | Focused on subjects with no invasive mechanical ventilation requierement |
| Behavioural activation therapy for depression in adults with non‐communicable diseases | 10.1002/14651858.CD013461.pub2 | Full review | Focused on subjects with no invasive mechanical ventilation requierement |
| Proton pump inhibitors for chronic obstructive pulmonary disease | 10.1002/14651858.CD013113.pub2 | Full review | Focused on subjects with no invasive mechanical ventilation requierement |
| Antenatal corticosteroids for accelerating fetal lung maturation for women at risk of preterm birth | 10.1002/14651858.CD004454.pub4 | Full review | Focused on subjects with no invasive mechanical ventilation requierement |
| Interventions to support the resilience and mental health of frontline health and social care professionals during and after a disease outbreak, epidemic or pandemic: a mixed methods systematic review | 10.1002/14651858.CD013779 | Full review | Focused on subjects with no invasive mechanical ventilation requierement |
| Corticosteroid therapy for nephrotic syndrome in children | 10.1002/14651858.CD001533.pub6 | Full review | Focused on subjects with no invasive mechanical ventilation requierement |
| Sublingual immunotherapy for asthma | 10.1002/14651858.CD011293.pub3 | Full review | Focused on subjects with no invasive mechanical ventilation requierement |
| Use of antimicrobial mouthwashes (gargling) and nasal sprays by healthcare workers to protect them when treating patients with suspected or confirmed COVID‐19 infection | 10.1002/14651858.CD013626.pub2 | Full review | Focused on subjects with no invasive mechanical ventilation requierement |
| Anti‐IL‐5 therapies for chronic obstructive pulmonary disease | 10.1002/14651858.CD013432.pub2 | Full review | Focused on subjects with no invasive mechanical ventilation requierement |
| Peripheral nerve blocks for hip fractures in adults | 10.1002/14651858.CD001159.pub3 | Full review | Focused on subjects with no invasive mechanical ventilation requierement |
| Corrector therapies (with or without potentiators) for people with cystic fibrosis with class II CFTR gene variants (most commonly F508del) | 10.1002/14651858.CD010966.pub3 | Full review | Focused on subjects with no invasive mechanical ventilation requierement |
| Respiratory muscle training for cystic fibrosis | 10.1002/14651858.CD006112.pub5 | Full review | Focused on subjects with no invasive mechanical ventilation requierement |
| Complementary and alternative therapies for post‐caesarean pain | 10.1002/14651858.CD011216.pub2 | Full review | Focused on subjects with no invasive mechanical ventilation requierement |
| Educational interventions for preventing lead poisoning in workers | 10.1002/14651858.CD013097.pub2 | Full review | Focused on subjects with no invasive mechanical ventilation requierement |
| Pneumococcal conjugate vaccines for preventing acute otitis media in children | 10.1002/14651858.CD001480.pub6 | Full review | Focused on subjects with no invasive mechanical ventilation requierement |
| Prophylactic anti‐staphylococcal antibiotics for cystic fibrosis | 10.1002/14651858.CD001912.pub5 | Full review | Focused on subjects with no invasive mechanical ventilation requierement |
| Drug treatment for spinal muscular atrophy types II and III | 10.1002/14651858.CD006282.pub5 | Full review | Focused on subjects with no invasive mechanical ventilation requierement |
| Topical treatment for facial burns | 10.1002/14651858.CD008058.pub3 | Full review | Focused on subjects with no invasive mechanical ventilation requierement |
| Continuous positive airway pressure (CPAP) for apnoea of prematurity | 10.1002/14651858.CD013660 | Protocol | Focused on subjects requiering CPAP |
| Preoperative fasting for prevention of perioperative complications in adults | 10.1002/14651858.CD013772 | Protocol | Focused on subjects requiering CPAP |
| Respiratory interventions for breathlessness in adults with advanced diseases | 10.1002/14651858.CD012683.pub2 | Protocol | Focused on subjects requiering CPAP |
| Prophylactic or very early initiation of continuous positive airway pressure (CPAP) for preterm infants | 10.1002/14651858.CD001243.pub4 | Full review | Focused on subjects requiering CPAP |
| Nasal continuous positive airway pressure levels for the prevention of morbidity and mortality in preterm infants | 10.1002/14651858.CD012778.pub2 | Full review | Focused on subjects requiering CPAP |
| Fluid restriction in the management of transient tachypnea of the newborn | 10.1002/14651858.CD011466.pub2 | Full review | Focused on subjects with no invasive mechanical ventilation requierement |
| High‐flow nasal cannulae for respiratory support in adult intensive care patients | 10.1002/14651858.CD010172.pub3 | Full review | Focused on subjects with no invasive mechanical ventilation requierement |
| Salbutamol for transient tachypnea of the newborn | 10.1002/14651858.CD011878.pub3 | Full review | Focused on subjects with no invasive mechanical ventilation requierement |
| Chloroquine or hydroxychloroquine for prevention and treatment of COVID‐19 | 10.1002/14651858.CD013587.pub2 | Full review | Focused on subjects with no invasive mechanical ventilation requierement |
| Chronic non‐invasive ventilation for chronic obstructive pulmonary disease | 10.1002/14651858.CD002878.pub3 | Full review | Focused on subjects requiering NIV |
| Non‐invasive positive airway pressure therapy for improving erectile dysfunction in men with obstructive sleep apnoea | 10.1002/14651858.CD013169.pub2 | Full review | Focused on subjects requiering NIV |
| Autogenic drainage for airway clearance in cystic fibrosis | 10.1002/14651858.CD009595.pub3 | Full review | Focused on subjects with no invasive mechanical ventilation requierement |
| Antenatal corticosteroids prior to planned caesarean at term for improving neonatal outcomes | 10.1002/14651858.CD006614.pub4 | Full review | Focused on subjects with no invasive mechanical ventilation requierement |
| Vitamin D supplementation for the treatment of COVID‐19: a living systematic review | 10.1002/14651858.CD015043 | Full review | Focused on subjects with no invasive mechanical ventilation requierement |
| Palivizumab for preventing severe respiratory syncytial virus (RSV) infection in children | 10.1002/14651858.CD013757.pub2 | Full review | Focused on subjects with no invasive mechanical ventilation requierement |
| Heliox for croup in children | 10.1002/14651858.CD006822.pub6 | Full review | Focused on subjects with no invasive mechanical ventilation requierement |
| Antioxidants to prevent respiratory decline in people with Duchenne muscular dystrophy and progressive respiratory decline | 10.1002/14651858.CD013720.pub3 | Full review | Focused on subjects with no invasive mechanical ventilation requierement |
| Pulmonary rehabilitation for interstitial lung disease | 10.1002/14651858.CD006322.pub4 | Full review | Focused on subjects with no invasive mechanical ventilation requierement |
| Interventions for palliative symptom control in COVID‐19 patients | 10.1002/14651858.CD015061 | Full review | Focused on subjects with no invasive mechanical ventilation requierement |
| Prehabilitation exercise therapy before elective abdominal aortic aneurysm repair | 10.1002/14651858.CD013662.pub2 | Full review | Focused on subjects with no invasive mechanical ventilation requierement |
| Cough augmentation techniques for people with chronic neuromuscular disorders | 10.1002/14651858.CD013170.pub2 | Full review | Focused on subjects with no invasive mechanical ventilation requierement |
| SARS‐CoV‐2‐neutralising monoclonal antibodies for treatment of COVID‐19 | 10.1002/14651858.CD013825.pub2 | Full review | Focused on subjects with no invasive mechanical ventilation requierement |
| Antibiotic regimens for early‐onset neonatal sepsis | 10.1002/14651858.CD013837.pub2 | Full review | Focused on subjects with no invasive mechanical ventilation requierement |
| Antibiotic regimens for late‐onset neonatal sepsis | 10.1002/14651858.CD013836.pub2 | Full review | Focused on subjects with no invasive mechanical ventilation requierement |
| Digital interventions for the management of chronic obstructive pulmonary disease | 10.1002/14651858.CD013246.pub2 | Full review | Focused on subjects with no invasive mechanical ventilation requierement |
| Anti‐interleukin‐13 and anti‐interleukin‐4 agents versus placebo, anti‐interleukin‐5 or anti‐immunoglobulin‐E agents, for people with asthma | 10.1002/14651858.CD012929.pub2 | Full review | Focused on subjects with no invasive mechanical ventilation requierement |
| Antibiotic therapy versus no antibiotic therapy for children aged 2 to 59 months with WHO‐defined non‐severe pneumonia and wheeze | 10.1002/14651858.CD009576.pub3 | Full review | Focused on subjects with no invasive mechanical ventilation requierement |
| Supervised maintenance programmes following pulmonary rehabilitation compared to usual care for chronic obstructive pulmonary disease | 10.1002/14651858.CD013569.pub2 | Full review | Focused on subjects with no invasive mechanical ventilation requierement |
| Enzyme replacement therapy with galsulfase for mucopolysaccharidosis type VI | 10.1002/14651858.CD009806.pub3 | Full review | Focused on subjects with no invasive mechanical ventilation requierement |
| Parenteral versus enteral fluid therapy for children hospitalised with bronchiolitis | 10.1002/14651858.CD013552.pub2 | Full review | Focused on subjects with no invasive mechanical ventilation requierement |
| Drug therapies for reducing gastric acidity in people with cystic fibrosis | 10.1002/14651858.CD003424.pub5 | Full review | Focused on subjects with no invasive mechanical ventilation requierement |
| Combination fixed‐dose beta agonist and steroid inhaler as required for adults or children with mild asthma | 10.1002/14651858.CD013518.pub2 | Full review | Focused on subjects with no invasive mechanical ventilation requierement |
| Prophylactic antibiotics for adults with chronic obstructive pulmonary disease: a network meta‐analysis | 10.1002/14651858.CD013198.pub2 | Full review | Focused on subjects with no invasive mechanical ventilation requierement |
| Telerehabilitation for chronic respiratory disease | 10.1002/14651858.CD013040.pub2 | Full review | Focused on subjects with no invasive mechanical ventilation requierement |
| Prophylactic antibiotics for preventing pneumococcal infection in children with sickle cell disease | 10.1002/14651858.CD003427.pub5 | Full review | Focused on subjects with no invasive mechanical ventilation requierement |
| Exercise training for bronchiectasis | 10.1002/14651858.CD013110.pub2 | Full review | Focused on subjects with no invasive mechanical ventilation requierement |
| Regular treatment with formoterol and an inhaled corticosteroid versus regular treatment with salmeterol and an inhaled corticosteroid for chronic asthma: serious adverse events | 10.1002/14651858.CD007694.pub3 | Full review | Focused on subjects with no invasive mechanical ventilation requierement |
| Timing of dornase alfa inhalation for cystic fibrosis | 10.1002/14651858.CD007923.pub6 | Full review | Focused on subjects with no invasive mechanical ventilation requierement |
| Wheat flour fortification with iron and other micronutrients for reducing anaemia and improving iron status in populations | 10.1002/14651858.CD011302.pub3 | Full review | Focused on subjects with no invasive mechanical ventilation requierement |
| Dornase alfa for cystic fibrosis | 10.1002/14651858.CD001127.pub5 | Full review | Focused on subjects with no invasive mechanical ventilation requierement |
| Antibiotics for treatment of sore throat in children and adults | 10.1002/14651858.CD000023.pub5 | Full review | Focused on subjects with no invasive mechanical ventilation requierement |
| Vaccines for measles, mumps, rubella, and varicella in children | 10.1002/14651858.CD004407.pub5 | Full review | Focused on subjects with no invasive mechanical ventilation requierement |
| Exercise training for adult lung transplant recipients | 10.1002/14651858.CD012307.pub2 | Full review | Focused on subjects with no invasive mechanical ventilation requierement |
| Siponimod for multiple sclerosis | 10.1002/14651858.CD013647.pub2 | Full review | Focused on subjects with no invasive mechanical ventilation requierement |
| Interventions for preventing distal intestinal obstruction syndrome (DIOS) in cystic fibrosis | 10.1002/14651858.CD012619.pub3 | Full review | Focused on subjects with no invasive mechanical ventilation requierement |
| Continuous glucose monitoring systems for monitoring cystic fibrosis‐related diabetes | 10.1002/14651858.CD013755.pub2 | Full review | Focused on subjects with no invasive mechanical ventilation requierement |
| Antibiotic therapy for chronic infection with Burkholderia cepacia complex in people with cystic fibrosis | 10.1002/14651858.CD013079.pub3 | Full review | Focused on subjects with no invasive mechanical ventilation requierement |
| Humidification of indoor air for preventing or reducing dryness symptoms or upper respiratory infections in educational settings and at the workplace | 10.1002/14651858.CD012219.pub2 | Full review | Focused on subjects with no invasive mechanical ventilation requierement |
| Interventions to improve adherence to pharmacological therapy for chronic obstructive pulmonary disease (COPD) | 10.1002/14651858.CD013381.pub2 | Full review | Focused on subjects with no invasive mechanical ventilation requierement |
| Telehealth interventions: remote monitoring and consultations for people with chronic obstructive pulmonary disease (COPD) | 10.1002/14651858.CD013196.pub2 | Full review | Focused on subjects with no invasive mechanical ventilation requierement |
| Vitamin C supplementation for prevention and treatment of pneumonia | 10.1002/14651858.CD013134.pub3 | Full review | Focused on subjects with no invasive mechanical ventilation requierement |
| Macrolides versus placebo for chronic asthma | 10.1002/14651858.CD002997.pub5 | Full review | Focused on subjects with no invasive mechanical ventilation requierement |
| Early warning systems and rapid response systems for the prevention of patient deterioration on acute adult hospital wards | 10.1002/14651858.CD005529.pub3 | Full review | Focused on subjects with no invasive mechanical ventilation requierement |
| Hybrid repair versus conventional open repair for aortic arch dissection | 10.1002/14651858.CD012920.pub2 | Full review | Focused on subjects with no invasive mechanical ventilation requierement |
| Lacosamide add‐on therapy for focal epilepsy | 10.1002/14651858.CD008841.pub3 | Full review | Focused on subjects with no invasive mechanical ventilation requierement |
| Single‐dose intravenous ketorolac for acute postoperative pain in adults | 10.1002/14651858.CD013263.pub2 | Full review | Focused on subjects with no invasive mechanical ventilation requierement |
| Pharmacological and surgical interventions for the treatment of gastro‐oesophageal reflux in adults and children with asthma | 10.1002/14651858.CD001496.pub2 | Full review | Focused on subjects with no invasive mechanical ventilation requierement |
| Individual‐level interventions to reduce personal exposure to outdoor air pollution and their effects on people with long‐term respiratory conditions | 10.1002/14651858.CD013441.pub2 | Full review | Focused on subjects with no invasive mechanical ventilation requierement |
| Physiological track‐and‐trigger/early warning systems for use in maternity care | 10.1002/14651858.CD013276.pub2 | Full review | Focused on subjects with no invasive mechanical ventilation requierement |
| Single versus combination intravenous anti‐pseudomonal antibiotic therapy for people with cystic fibrosis | 10.1002/14651858.CD002007.pub5 | Full review | Focused on subjects with no invasive mechanical ventilation requierement |
| Integrated disease management interventions for patients with chronic obstructive pulmonary disease | 10.1002/14651858.CD009437.pub3 | Full review | Focused on subjects with no invasive mechanical ventilation requierement |
| Renin‐angiotensin system inhibitors for treating COVID‐19 in adults | 10.1002/14651858.CD013831 | Protocol | Did not specify invasive mechanical ventilation requierement when defining population |
| Vitamin A for preventing acute lower respiratory tract infections in children up to seven years of age | 10.1002/14651858.CD014847 | Protocol | Focused on subjects requiering CPAP |
| Preoperative fasting for prevention of perioperative complications in children | 10.1002/14651858.CD013809.pub2 | Protocol | Focused on subjects requiering CPAP |
| Bicarbonate for acute acidosis | 10.1002/14651858.CD014371 | Protocol | Did not specify invasive mechanical ventilation requierement when defining population |
| Post‐extubation use of non‐invasive respiratory support in preterm infants: a network meta‐analysis | 10.1002/14651858.CD014509 | Protocol | Focused on subjects requiering NIV |
| Non‐invasive respiratory support in preterm infants as primary mode: a network meta‐analysis | 10.1002/14651858.CD014895 | Protocol | Focused on subjects requiering NIV |
| Pharmacological interventions for improving respiratory symptoms and function in amyotrophic lateral sclerosis | 10.1002/14651858.CD010030.pub2 | Protocol | Focused on subjects requiering CPAP |
| Respiratory muscle training for obstructive sleep apnoea | 10.1002/14651858.CD015039 | Protocol | Focused on subjects requiering CPAP |
| Manual ventilation devices for neonatal resuscitation | 10.1002/14651858.CD004949.pub2 | Protocol | Focused on subjects requiering CPAP |
| Continuous positive airway pressure (CPAP) for acute bronchiolitis in children | 10.1002/14651858.CD010473.pub4 | Full review | Focused on subjects requiering CPAP |
| Ultrafiltration for acute heart failure | 10.1002/14651858.CD013593.pub2 | Full review | Focused on subjects with no invasive mechanical ventilation requierement |
| Ivermectin for preventing and treating COVID‐19 | 10.1002/14651858.CD015017.pub3 | Full review | Focused on subjects with no invasive mechanical ventilation requierement |
| Masks versus prongs as interfaces for nasal continuous positive airway pressure in preterm infants | 10.1002/14651858.CD015129 | Full review | Focused on subjects requiering CPAP |
| Magnesium sulfate for acute exacerbations of chronic obstructive pulmonary disease | 10.1002/14651858.CD013506.pub2 | Full review | Focused on subjects with no invasive mechanical ventilation requierement |
| Non‐invasive positive pressure ventilation for central sleep apnoea in adults | 10.1002/14651858.CD012889.pub2 | Full review | Focused on subjects requiering NIV |
| Non‐pharmacological interventions for sleep promotion in hospitalized children | 10.1002/14651858.CD012908.pub2 | Full review | Focused on subjects with no invasive mechanical ventilation requierement |
| Exercise versus airway clearance techniques for people with cystic fibrosis | 10.1002/14651858.CD013285.pub2 | Full review | Focused on subjects with no invasive mechanical ventilation requierement |
| Treatment for sialorrhea (excessive saliva) in people with motor neuron disease/amyotrophic lateral sclerosis | 10.1002/14651858.CD006981.pub3 | Full review | Focused on subjects with no invasive mechanical ventilation requierement |
| Inhaled corticosteroids for the treatment of COVID‐19 | 10.1002/14651858.CD015125 | Full review | Focused on subjects with no invasive mechanical ventilation requierement |
| Fluvoxamine for the treatment of COVID‐19 | 10.1002/14651858.CD015391 | Full review | Focused on subjects with no invasive mechanical ventilation requierement |
| Effectiveness and tolerability of dual and triple combination inhaler therapies compared with each other and varying doses of inhaled corticosteroids in adolescents and adults with asthma: a systematic review and network meta‐analysis | 10.1002/14651858.CD013799.pub2 | Full review | Focused on subjects with no invasive mechanical ventilation requierement |
| Cervical pessary for preventing preterm birth in singleton pregnancies | 10.1002/14651858.CD014508 | Full review | Focused on subjects with no invasive mechanical ventilation requierement |
| Probiotics for preventing acute upper respiratory tract infections | 10.1002/14651858.CD006895.pub4 | Full review | Focused on subjects with no invasive mechanical ventilation requierement |
| Maternal postures for fetal malposition in labour for improving the health of mothers and their infants | 10.1002/14651858.CD014615 | Full review | Focused on subjects with no invasive mechanical ventilation requierement |
| Workplace interventions to reduce the risk of SARS‐CoV‐2 infection outside of healthcare settings | 10.1002/14651858.CD015112.pub2 | Full review | Focused on subjects with no invasive mechanical ventilation requierement |
| Tocolytics for delaying preterm birth: a network meta‐analysis (0924) | 10.1002/14651858.CD014978.pub2 | Full review | Focused on subjects with no invasive mechanical ventilation requierement |
| Interventions for reducing inflammation in familial Mediterranean fever | 10.1002/14651858.CD010893.pub4 | Full review | Focused on subjects with no invasive mechanical ventilation requierement |
| Inhaled bronchodilators for acute chest syndrome in people with sickle cell disease | 10.1002/14651858.CD003733.pub5 | Full review | Focused on subjects with no invasive mechanical ventilation requierement |
| Interventions for the eradication of meticillin‐resistant Staphylococcus aureus (MRSA) in people with cystic fibrosis | 10.1002/14651858.CD009650.pub5 | Full review | Focused on subjects with no invasive mechanical ventilation requierement |
| Perioperative pharmacological interventions for fetal immobilisation during fetal surgery and invasive procedures | 10.1002/14651858.CD011068.pub2 | Full review | Focused on subjects with no invasive mechanical ventilation requierement |
| Self‐management interventions for people with chronic obstructive pulmonary disease | 10.1002/14651858.CD002990.pub4 | Full review | Focused on subjects with no invasive mechanical ventilation requierement |
| Short‐acting inhaled bronchodilators for cystic fibrosis | 10.1002/14651858.CD013666.pub2 | Full review | Focused on subjects with no invasive mechanical ventilation requierement |
| Digital interventions to improve adherence to maintenance medication in asthma | 10.1002/14651858.CD013030.pub2 | Full review | Focused on subjects with no invasive mechanical ventilation requierement |
| Pulmonary rehabilitation versus usual care for adults with asthma | 10.1002/14651858.CD013485.pub2 | Full review | Focused on subjects with no invasive mechanical ventilation requierement |
| Preprocedural mouth rinses for preventing transmission of infectious diseases through aerosols in dental healthcare providers | 10.1002/14651858.CD013826.pub2 | Full review | Focused on subjects with no invasive mechanical ventilation requierement |
| Ocrelizumab for multiple sclerosis | 10.1002/14651858.CD013247.pub2 | Full review | Focused on subjects with no invasive mechanical ventilation requierement |
| Vitamin A supplementation for preventing morbidity and mortality in children from six months to five years of age | 10.1002/14651858.CD008524.pub4 | Full review | Focused on subjects with no invasive mechanical ventilation requierement |
| Videolaryngoscopy versus direct laryngoscopy for adults undergoing tracheal intubation | 10.1002/14651858.CD011136.pub3 | Full review | Focused on subjects undergoing surgery |
| Repeat doses of prenatal corticosteroids for women at risk of preterm birth for improving neonatal health outcomes | 10.1002/14651858.CD003935.pub5 | Full review | Focused on subjects with no invasive mechanical ventilation requierement |
| Medical interventions for chronic rhinosinusitis in cystic fibrosis | 10.1002/14651858.CD012979.pub3 | Full review | Focused on subjects with no invasive mechanical ventilation requierement |
| Physical activity and exercise training in cystic fibrosis | 10.1002/14651858.CD002768.pub5 | Full review | Focused on subjects with no invasive mechanical ventilation requierement |
| Different corticosteroids and regimens for accelerating fetal lung maturation for babies at risk of preterm birth | 10.1002/14651858.CD006764.pub4 | Full review | Focused on subjects with no invasive mechanical ventilation requierement |
| Systemic interventions for treatment of Stevens‐Johnson syndrome (SJS), toxic epidermal necrolysis (TEN), and SJS/TEN overlap syndrome | 10.1002/14651858.CD013130.pub2 | Full review | Focused on subjects with no invasive mechanical ventilation requierement |
| Educational interventions for health professionals managing chronic obstructive pulmonary disease in primary care | 10.1002/14651858.CD012652.pub2 | Full review | Focused on subjects with no invasive mechanical ventilation requierement |
| Intermittent prophylactic antibiotics for bronchiectasis | 10.1002/14651858.CD013254.pub2 | Full review | Focused on subjects with no invasive mechanical ventilation requierement |
| Inhaled anti‐pseudomonal antibiotics for long‐term therapy in cystic fibrosis | 10.1002/14651858.CD001021.pub4 | Full review | Focused on subjects with no invasive mechanical ventilation requierement |
| Immunostimulants versus placebo for preventing exacerbations in adults with chronic bronchitis or chronic obstructive pulmonary disease | 10.1002/14651858.CD013343.pub2 | Full review | Focused on subjects with no invasive mechanical ventilation requierement |
| Increased versus stable doses of inhaled corticosteroids for exacerbations of chronic asthma in adults and children | 10.1002/14651858.CD007524.pub5 | Full review | Focused on subjects with no invasive mechanical ventilation requierement |
| Biomarkers as point‐of‐care tests to guide prescription of antibiotics in people with acute respiratory infections in primary care | 10.1002/14651858.CD010130.pub3 | Full review | Focused on subjects with no invasive mechanical ventilation requierement |
| Family‐centred interventions for Indigenous early childhood well‐being by primary healthcare services | 10.1002/14651858.CD012463.pub2 | Full review | Focused on subjects with no invasive mechanical ventilation requierement |
| Vaccines for the common cold | 10.1002/14651858.CD002190.pub6 | Full review | Focused on subjects with no invasive mechanical ventilation requierement |
| Anti‐IL‐5 therapies for asthma | 10.1002/14651858.CD010834.pub4 | Full review | Focused on subjects with no invasive mechanical ventilation requierement |
| Efficacy and safety of COVID‐19 vaccines | 10.1002/14651858.CD015477 | Full review | Focused on subjects with no invasive mechanical ventilation requierement |
| Homeopathic medicinal products for preventing and treating acute respiratory tract infections in children | 10.1002/14651858.CD005974.pub6 | Full review | Focused on subjects with no invasive mechanical ventilation requierement |
| High versus low blood pressure targets for cardiac surgery while on cardiopulmonary bypass | 10.1002/14651858.CD013494.pub2 | Full review | Focused on subjects undergoing surgery |
| Respiratory syncytial virus vaccination during pregnancy for improving infant outcomes | 10.1002/14651858.CD015134 | Protocol | Focused on subjects with no invasive mechanical ventilation requierement |
| Prone position for management of respiratory failure in non‐intubated adults | 10.1002/14651858.CD014828 | Protocol | Focused on subjects with no invasive mechanical ventilation requierement |
| Vitamin D for preventing acute respiratory infections in children up to five years of age | 10.1002/14651858.CD015111 | Protocol | Focused on subjects with no invasive mechanical ventilation requierement |
| Adenoidectomy for otitis media with effusion (OME) in children | 10.1002/14651858.CD015252 | Protocol | Focused on subjects requiering CPAP |
| Ventilation tubes (grommets) for otitis media with effusion (OME) in children | 10.1002/14651858.CD015215 | Protocol | Focused on subjects requiering CPAP |
| Active cycle of breathing technique for cystic fibrosis | 10.1002/14651858.CD007862.pub5 | Full review | Focused on subjects with no invasive mechanical ventilation requierement |
| Risk of thromboembolism in patients with COVID‐19 who are using hormonal contraception | 10.1002/14651858.CD014908.pub2 | Full review | Focused on subjects with no invasive mechanical ventilation requierement |
| Digital technology for monitoring adherence to inhaled therapies in people with cystic fibrosis | 10.1002/14651858.CD013733.pub2 | Full review | Focused on subjects with no invasive mechanical ventilation requierement |
| Physical interventions to interrupt or reduce the spread of respiratory viruses | 10.1002/14651858.CD006207.pub6 | Full review | Focused on subjects with no invasive mechanical ventilation requierement |
| Vitamin D for the management of asthma | 10.1002/14651858.CD011511.pub3 | Full review | Focused on subjects with no invasive mechanical ventilation requierement |
| Antibiotics for chronic pulmonary infection in children with a neurodisability (neurodevelopmental disorder) | 10.1002/14651858.CD013813.pub2 | Full review | Focused on subjects with no invasive mechanical ventilation requierement |
| Glucocorticoids for croup in children | 10.1002/14651858.CD001955.pub5 | Full review | Focused on subjects with no invasive mechanical ventilation requierement |
| Inspiratory muscle training, with or without concomitant pulmonary rehabilitation, for chronic obstructive pulmonary disease (COPD) | 10.1002/14651858.CD013778.pub2 | Full review | Focused on subjects with no invasive mechanical ventilation requierement |
| Vitamin D as an adjunct to antibiotics for the treatment of acute childhood pneumonia | 10.1002/14651858.CD011597.pub3 | Full review | Focused on subjects with no invasive mechanical ventilation requierement |
| Oral vitamin A supplements to prevent acute upper respiratory tract infections in children up to seven years of age | 10.1002/14651858.CD015306 | Protocol | Focused on subjects with no invasive mechanical ventilation requierement |
